# Simulations indicate that scores of lionfish (*Pterois volitans*) colonized the Atlantic Ocean

**DOI:** 10.7717/peerj.3996

**Published:** 2017-12-19

**Authors:** Jason D. Selwyn, John E. Johnson, Alan M. Downey-Wall, Adam M. Bynum, Rebecca M. Hamner, J. Derek Hogan, Christopher E. Bird

**Affiliations:** 1 HoBi Lab, Department of Life Sciences, Texas A&M University—Corpus Christi, Corpus Christi, TX, United States of America; 2 Marine Science Center, Northeastern University, Nahant, MA, United States of America; 3 Hawai‘i Institute of Marine Biology, University of Hawai‘i at Mānoa, Kāne‘ohe, Hawai‘i, United States of America

**Keywords:** Alien invasive species, Invasion route, Gene surfing

## Abstract

The invasion of the western Atlantic Ocean by the Indo-Pacific red lionfish (*Pterois volitans*) has had devastating consequences for marine ecosystems. Estimating the number of colonizing lionfish can be useful in identifying the introduction pathway and can inform policy decisions aimed at preventing similar invasions. It is well-established that at least ten lionfish were initially introduced. However, that estimate has not faced probabilistic scrutiny and is based solely on the number of haplotypes in the maternally-inherited mitochondrial control region. To rigorously estimate the number of lionfish that were introduced, we used a forward-time, Wright-Fisher, population genetic model in concert with a demographic, life-history model to simulate the invasion across a range of source population sizes and colonizing population fecundities. Assuming a balanced sex ratio and no Allee effects, the simulations indicate that the Atlantic population was founded by 118 (54–514, 95% HPD) lionfish from the Indo-Pacific, the Caribbean by 84 (22–328, 95% HPD) lionfish from the Atlantic, and the Gulf of Mexico by at least 114 (no upper bound on 95% HPD) lionfish from the Caribbean. Increasing the size, and therefore diversity, of the Indo-Pacific source population and fecundity of the founding population caused the number of colonists to decrease, but with rapidly diminishing returns. When the simulation was parameterized to minimize the number of colonists (high *θ* and relative fecundity), 96 (48–216, 95% HPD) colonists were most likely. In a more realistic scenario with Allee effects (e.g., 50% reduction in fecundity) plaguing the colonists, the most likely number of lionfish increased to 272 (106–950, 95% HPD). These results, in combination with other published data, support the hypothesis that lionfish were introduced to the Atlantic via the aquarium trade, rather than shipping. When building the model employed here, we made assumptions that minimize the number of colonists, such as the lionfish being introduced in a single event. While we conservatively modelled the introduction pathway as a single release of lionfish in one location, it is more likely that a combination of smaller and larger releases from a variety of aquarium trade stakeholders occurred near Miami, Florida, which could have led to even larger numbers of colonists than simulated here. Efforts to prevent future invasions via the aquarium trade should focus on the education of stakeholders and the prohibition of release, with adequate rewards for compliance and penalties for violations.

## Introduction

The ability to successfully eradicate an alien-invasive species is directly correlated with the population size (Epanchin-Niell & Hastings, 2010) and the amount of time that has passed since the initial introduction and establishment (see Blackburn et al., 2011). If the alien population becomes invasive and spreads after establishing, eradication becomes nearly impossible without vast financial commitments (Epanchin-Niell & Hastings, 2010). Preventative measures and early detection enable the effective control of alien species before exponential population growth exceeds conventional control methods (Jarrad et al., 2011). Given the costs of control after a species becomes invasive, prevention of future invasions is the most effective approach to avoid the destruction of ecosystems and protect economic interests (CBD, 2002; Pimentel, Zuniga & Morrison, 2005; Simberloff et al., 2013).

Elucidating the likely route of transport for established and spreading alien species, and determining how many colonists established the initial population can be used to inform preventative management and education efforts (Estoup & Guillemaud, 2010; Hulme, 2015; McGeoch et al., 2016). While the initial mechanism of introduction is rarely conclusively identified (Brockerhoff et al., 2014), understanding the number of invaders and the frequency of introductions (i.e., propagule pressure) can be used to infer likely invasion routes (Hulme, 2015). Propagule pressure is directly correlated with the ability of an introduced species to become established and spread (Yang et al., 2012; Brockerhoff et al., 2014). To determine the propagule pressure and infer likely invasion routes, it is important to have probabilistic estimates of the number of invaders initially present (García-Díaz et al., 2015).

Genetic methodologies are particularly useful in reconstructing the history of introduced species (Estoup & Guillemaud, 2010; Rius et al., 2015; Cristescu, 2015). For example, biological invasions that occur following an initial population bottleneck will typically exhibit founder effects, which can be exploited to estimate the initial number of founders (Azzurro et al., 2006; Golani et al., 2007). Both fewer colonists and fewer introduction events can result in more severe genetic bottlenecks (Roman & Darling, 2007). Following colonization, additional genetic diversity is likely to be lost in small populations due to strong genetic drift (Allendorf, 1986). Integrated population genetic (Fisher, 1922; Wright, 1931) and demographic models can be used to infer and predict founding population sizes and diversity (Ficetola, Bonin & Miaud, 2008; Hulme et al., 2008; Tran, Hofrichter & Jost, 2012; Benson et al., 2016).

The invasion of the western Atlantic Ocean by Indo-Pacific lionfish (*Pterois volitans, P. miles*) has been recognized as a global environmental problem (Sutherland et al., 2010; Hixon et al., 2016). In the Atlantic, alien-invasive lionfish consume both economically and ecologically important species (Morris & Akins, 2009; Côté et al., 2013). The net effect of adding lionfish to the ecosystem has been a reduction in recruitment and biomass of native species that fall prey to the lionfish. In some cases severe reductions have been observed(Albins & Hixon, 2008; Green et al., 2012; Selwyn et al., 2014; however, see Hackerott et al., 2017). Lionfish have also been indirectly tied to a shift from coral to algal-dominated habitats through the consumption of herbivorous fishes (Albins & Hixon, 2011; Lesser & Slattery, 2011). Given their range and population size, conventional eradication is not a viable option for controlling these effects at a regional scale (Côté, Green & Hixon, 2013), despite the efficacy of local management in mitigating local effects (Green et al., 2014; Usseglio et al., 2017). However, there is some evidence for a natural decline in invasive lionfish abundance in the Bahamas from 2011 to 2015 (Benkwitt et al., 2017).

Lionfish are believed to have been transported from the Indo-Pacific to the Atlantic via the aquarium trade (Hare & Whitfield, 2003; Semmens et al., 2004; Ruiz-Carus et al., 2006; Morris & Whitfield, 2009). The initial sightings of lionfish in the western Atlantic occurred in Florida in the mid-1980s (Courtenay, 1995; Morris & Akins, 2009). Population growth was slow following detection (Whitfield et al., 2002), an indication of low fecundity or high mortality possibly due to Allee effects (the suite of phenomena that have negative effects on fitness and growth in small populations; Allee & Bowen (1932); Taylor & Hastings, 2005; Tobin et al., 2007). By 2007, however, the invasion was characterized by large populations (Whitfield et al., 2007) with exponential growth (Green et al., 2012). Between 2007 and 2010, lionfish spread across the Caribbean (Schofield, 2009; Betancur et al., 2011). In 2010 the invasion front entered the Gulf of Mexico, originating from the Caribbean population (Schofield, 2010; Johnson et al., 2016). The stepping-stone pattern of this invasion has resulted in three genetically distinct lionfish populations, one in each of these regions, with each subsequently colonized region exhibiting progressively fewer haplotypes (Johnson et al., 2016).

The minimum number of *P. volitans* that colonized the Atlantic has been estimated to be between six and 10 (Betancur et al., 2011); however, this estimate is based upon counts of unique haplotypes from a 674 bp fragment of mtDNA and is not a robust reflection of the number of lionfish that were introduced. Using the number of observed haplotypes to estimate the number of introduced lionfish is not rooted in a rigorous probabilistic framework that considers the genetic compositions of both the source and invasive populations. Therefore, this estimate of 6-10 colonizing lionfish is not useful to inform either a further understanding of the processes leading to the establishment of the invasion or policy aimed at preventing similar future invasions.

The lower estimate of six colonists involves the assumption that four haplotypes have originated through mutation in the Atlantic, but this is unlikely. The behavior of allelic diversity in expanding populations is well established (Nei, Maruyama & Chakraborty, 1975; Maruyama & Fuerst, 1985), and is characterized by an excess of rare alleles. However, a genetic pattern of population expansion takes time to develop because mutation rates, even in mitochondrial DNA, are relatively slow (see Lynch, 2010). The invasive lionfish population has yet to exhibit the characteristic genetic signature of an expanding population, despite a large documented population size and widespread expansion. Thus, it is exceedingly unlikely that any of the haplotypes observed in the Atlantic are the result of mutations that arose in the Atlantic. Indeed, in the well-documented invasion of Hawai‘i by the bluestripe snapper (*Lutjanus kasmira*), which predates the Atlantic lionfish invasion by roughly 20 years, no signature of population expansion is evident in the mitochondrial control region either (Gaither et al., 2010).

If 10 *P. volitans* colonized the western Atlantic, it is consistent with the hypothesis one, or a few, that releases by home aquarists, distributors, or other aquarium trade intermediaries could have triggered the invasion. The hypothesis that only 10 lionfish were introduced is tenuous, however, because it does not account for the genetic diversity of the source population or the frequencies of haplotypes in the Atlantic. We hypothesized that when considering additional available genetic information, it was highly improbable that only one male and nine female lionfish (with nine unique haplotypes) established the Atlantic population. If many more lionfish colonized the Atlantic, then it could indicate that the scale of releases was larger than previously thought, and that home aquarists, alone, are insufficient to explain the pathway by which lionfish were introduced to the Atlantic.

Here we simulate the lionfish (*P. volitans*) invasion and conservatively estimate the number of lionfish that colonized the western Atlantic Ocean, Caribbean, and Gulf of Mexico using a coupled demographic-population genetic model. The results of the simulation are used to re-evaluate the mechanism by which lionfish were introduced to the Atlantic and how this affects the management of future invasions.

## Methods

### Characterizing genetic composition of wild populations

A total of 1,294 mitochondrial control region sequences consisting of 30 unique haplotypes were gathered from GenBank and published literature (Table 1; Freshwater et al., 2009; Betancur et al., 2011; Toledo-Hernández et al., 2014; Butterfield et al., 2015; Johnson et al., 2016). The sequences were obtained from *P. volitans* in Indonesia and the western Atlantic Ocean. Johnson et al. (2016) report that lionfish in the western Atlantic exhibit genetic structure among the western North Atlantic, Caribbean, and the Gulf of Mexico, but not within these regions. Consequently, sampling locations within each of these three regions were pooled for this analysis.

**Table 1:** Source material and population summaries. *Pterois volitans* mitochondrial d-loop haplotype data utilized in the present study with summaries of haplotype richness and diversity.

Region	Number of individuals	Haplotype richness	Haplotype diversity (95% CI)	References	Accession numbers
Indonesia	36	21	0.97 (0.95–1.00)	1	FJ516418 –FJ516438
North Atlantic	459	9	0.67 (0.64–0.70)	1, 2, 4	FJ516409, FJ516410, FJ516411, FJ516412, FJ516413, FJ516414, FJ516415, FJ516416, FJ516417
Caribbean	601	4	0.47 (0.43–0.50)	2–4	FJ516409, FJ516410, FJ516411, FJ516412
Gulf of Mexico	188	3	0.55 (0.49–0.59)	5	FJ516409, FJ516410, FJ516412

**Notes.**

1, Freshwater et al. (2009), 2, Betancur et al. (2011), 3, Toledo-Hernández et al. (2014), 4, Butterfield et al. (2015) and 5, Johnson et al. (2016).

Because the Indo-Pacific population that was the source of the Atlantic lionfish invasion has not been sampled, we needed to estimate its genetic composition. We know that the source population has not been sampled because none of the lionfish control region haplotypes in the western Atlantic have been sampled anywhere else. If the source population is in equilibrium and the mitochondrial control region conforms with the infinite sites model, then the population parameter, *θ*, is tightly associated with the genetic composition of the population. In this case, Ewens’ (1972) sampling formula can be used to simulate the sampling of alleles from populations knowing only *θ* = 2*N*_*e*_*μ*, where *N*_*e*_ is the effective population size and *μ* is the mutation rate (Hartl & Clark, 2006). We used the mean number of pairwise differences and the observed number of segregating sites in a population sample of Indonesian lionfish (Freshwater et al., 2009) to estimate the population parameter, *θ* and its standard deviation (*s*) using Arlequin 3.5 (Excoffier & Lischer, 2010). To determine whether the mitochondrial control region in populations of Indo-Pacific lionfish conforms with the infinite alleles and sites models of nucleotide evolution (Kimura, 1969; Watterson, 1975; Tajima, 1996), which are nearly identical for linked nucleotides like those in the mitochondrial control region (Hartl & Clark, 2006), we performed the Ewens–Watterson test (Ewens, 1972; Watterson, 1978), Tajima’s D (Tajima, 1989), and Fu’s *F*_*s*_ (Fu, 1997) based on 10,000 simulations in Arlequin.

Haplotype richness and diversity were estimated in the wild alien-invasive lionfish populations of the western North Atlantic, Caribbean, and the Gulf of Mexico, so they could be compared with simulated invading populations. Haplotype richness was calculated as the number of unique haplotypes that have been observed in each population. Haplotype diversity was calculated as the probability of drawing two different haplotypes at random from the population with 95% confidence intervals calculated based on 1,000 bootstraps sampled with replacement (Nei & Tajima, 1981).

### Simulating colonization

The colonizations of the western North Atlantic, Caribbean, and Gulf of Mexico were each simulated as single introduction events of female lionfish. The record of lionfish sightings in the western North Atlantic is consistent with lionfish being introduced near Miami, Florida (Schofield, 2009). Modelling the colonizations as single introduction events minimizes the estimated number of colonists because, relative to scenarios with multiple colonization events from the same source population with fewer lionfish per event, (1) the effect of genetic drift removing diversity from the population is minimized by the maximized initial population size, and (2) Allee effects are minimized by increased mating opportunities (Roman & Darling, 2007). Consequently, when compared to a single colonization event, multiple colonization events from the same source population would require no fewer, and likely more, individuals for the establishment of invasive populations with the observed allelic richness and mitochondrial haplotype diversity.

The number of introduced female lionfish was varied from 1 to 800. We chose to model only females because the best available genetic data from lionfish in the western Atlantic is maternally transmitted mitochondrial DNA. The model we employ assumes that enough males colonize to fertilize the eggs of the females. We also assumed that the females are adults. While it is likely that the Atlantic was colonized by adult lionfish from the Indo-Pacific, we acknowledge that the Caribbean and Gulf of Mexico were likely to be colonized by larvae. It is not our goal, however, to estimate the number of larvae that colonized the Caribbean and Gulf of Mexico. Rather, it is our goal to estimate the number of adult females that founded these populations.

Females were sampled from either (1) an Indo-Pacific population in mutation—drift equilibrium that conforms to the infinite alleles mutational model or (2) Atlantic and Caribbean populations with the same allele frequency distribution observed in extant empirical samples. The Indo-Pacific population was characterized by the population genetic parameter *θ* for a sample of Indonesian lionfish, and samples were generated using Ewens’ (1972) sampling formula (Crane, 2016). For the Atlantic and Caribbean source populations which are too new to be in mutation-drift equilibrium and have been identified as the source populations, the R function, rmultinom, was used to generate samples from the observed multinomial distribution of sampled haplotype frequencies in the western North Atlantic and Caribbean populations when simulating the invasions in the Caribbean and Gulf of Mexico, respectively.

To test for model sensitivity to error in the estimate of *θ* (greater or lesser genetic diversity or more source populations), simulations assuming the source population was characterized by *θ* ± *s* and *θ* ± 2*s* were also run, where *s* is the standard deviation of the estimate of *θ*.

#### Demographic model

An individual-based model adapted from the stage-based matrix model developed by Morris, Shertzer & Rice (2011) was used to simulate lionfish population growth (Downey-Wall, 2016). The model was initialized with colonizing adult females, which minimizes the number of colonists required to begin an invasion, relative to the introduction of larvae or juveniles. The model proceeded in monthly time-steps and divides the lionfish life history into three primary life stages (larvae, juvenile, and adult). We further divided the juvenile life stage into 11 juvenile stages of one month to appropriately simulate the delay in the reproductive maturity of Atlantic-derived lionfish, which would not be accurately simulated by the model of Morris, Shertzer & Rice (2011). Life-stage-specific demographic parameters from lionfish were used, as outlined by Morris, Shertzer & Rice (2011; also see for additional information regarding the estimation and sensitivity of these parameters).

Briefly, adult fecundity (194,577 eggs/month/adult) was calculated from the reproductive contribution (number of eggs) per female per spawn (*R*_*A*_ = 35,315, Morris, 2009) and the spawning rate of 7.9/month/female (Morris, 2009; for confirmation see Gardner et al., 2015). The eggs had a mortality rate of 0.31/day (McGurk, 1987) before hatching after three days (Morris, 2009). The larval stage lasted for the rest of the month (Ahrenholz & Morris, 2010) with a mortality rate of 0.35/day (McGurk, 1987). Ultimately, the proportion of larvae that survived and metamorphosed into juveniles was 0.00003 (*G*_*L*_). The 11 juvenile stages had a mortality rate of 0.165/month (*m*_*J*_). The adult life span was controlled by mortality rate, which was 0.052/month (*m*_*A*_, Lorenzen, 1996).

To simulate the effect of a slower growth rate at the outset of the invasion due to Allee effects caused by low population densities (Stephens, Sutherland & Freckleton, 1999), we adjusted the monthly fecundity to 25%, 50%, 75%, and 100% (relative fecundity, *r*) of that reported by Morris, Shertzer & Rice (2011). Allee effects can reduce the fecundity to 25–50%, or lower, in natural populations (Roll et al., 1997; Morgan, 1999; Berec, Angulo & Courchamp, 2007).

A series of four discrete, stage-based equations were used to calculate the number of individuals for each haplotype at each subsequent time point (*t* + 1) from the number at time point (*t*; set in monthly time-steps):

(1) \begin{eqnarray*}& & {n}_{L}(t+1)=r{R}_{A}{n}_{A}(t)\end{eqnarray*}

(2) \begin{eqnarray*}& & {n}_{J,1}(t+1)={G}_{L}{n}_{L}(t)+(1-{m}_{J}){n}_{J,1}(t)\end{eqnarray*}

(3) \begin{eqnarray*}& & {n}_{J,s+1}(t+1)=(1-{m}_{J}){n}_{J,s}(t)1\lt s\lt 11\end{eqnarray*}

(4) \begin{eqnarray*}& & {n}_{A}(t+1)=(1-{m}_{J}){n}_{J,11}(t)+(1-{m}_{A}){n}_{A}(t)\end{eqnarray*}

where *n*_[*L*,*J*,*A*]_ represent the number of individuals at the larval, juvenile (*s* represents the 10, monthly juvenile stages), and adult stages, respectively (other variables are defined in the description of the demographic model, above). The model was run for 15 years to encompass the time from the initial lionfish sighting to range expansion (Whitfield et al., 2002).

#### Genetic drift model

The demographic growth model was used to determine the number of larvae and adults in a Wright-Fisher, forward-time model of genetic drift with overlapping generations (Fisher, 1922; Wright, 1931). Each month, haplotypes are sampled with replacement from the pool of eggs produced by the adult females to create the new cohort of lionfish larvae. This model assumes a mutation rate of zero due to the short time scale of the invasion, and there is no evidence of mutations driving mtDNA diversity in western Atlantic lionfish populations. Running the model for 15 years was sufficient time for haplotype frequencies to stabilize without mutation due to decreased genetic drift with increasing population size.

### Assessing simulations

Following the simulation, a sample was drawn from the simulated population that was equal in size to the number sampled and reported by researchers in the destination population. For example, 459 lionfish have been sampled from the western North Atlantic (Freshwater et al., 2009; Betancur et al., 2011; Butterfield et al., 2015; Johnson et al., 2016), therefore 459 were sampled from the simulated western North Atlantic population. The haplotype richness and diversity of the simulated invasive populations were calculated for each simulation in the same manner as was calculated for the observed population. The conditional joint probability of finding the observed haplotype richness and haplotype diversity within the 95% confidence interval given the number of female colonists was calculated based on 10,000 simulations. We calculated the 50% and 95% highest probability density interval (HPD) of the initial number of females using the CODA package (Plummer et al., 2006). We chose to use the HPD given the skewed nature of the distribution with an extremely sharp lower bound and a generally long and trailing right-hand tail (Gelman et al., 2013). Note that in some cases, the HPD estimates will be skewed downwards by our choice to limit the number of female colonists to a maximum of 800, but in these cases our major point is that the estimates are much larger than nine colonists and there is no qualitative alteration of conclusions. This process was repeated for each source and destination population.

All above analyses were performed in R v 3.3.1 using code written by the authors, with figures made using the package GGPLOT2 (Supplement B, Wickham, 2009; R Core Team, 2015).

## Results

### Genetic composition and diversity of wild populations

Based on the mitochondrial control region haplotypes present in Indonesia, *θ* was estimated to be 7.64 (±2.73 SD). Two of the three tests employed indicate that the population in Indonesia was consistent with the assumptions of the infinite alleles and sites models (Tajima’s *D* =  − 1.161, *p* = 0.111; Fu’s *F* =  − 8.008, *p* = 0.004; Ewens–Watterson test *F* = 0.077, *p* = 0.0807), thereby indicating that Ewens’ (1972) sampling formula, parameterized with *θ*, provides a valid methodology for sampling the Indo-Pacific source population.

The invasive lionfish populations in the western North Atlantic, Caribbean and Gulf of Mexico exhibited haplotype richnesses of 9, 4, and 3, respectively with associated haplotype diversity estimated as 0.67, 0.47, and 0.55, respectively (Table 1). These values were used to compare simulated colonizing populations with observed wild populations to assess the most probable number of colonists. Note that the Gulf of Mexico has slightly higher haplotype diversity than the Caribbean despite being derived from the Caribbean and having fewer haplotypes. This is either a result of slightly different allele frequencies caused by genetic drift during the colonization of the Gulf or statistical sampling error (see discussion of gene surfing in Johnson et al., 2016).

### Indo-Pacific to western North Atlantic simulation

**Figure 1:**
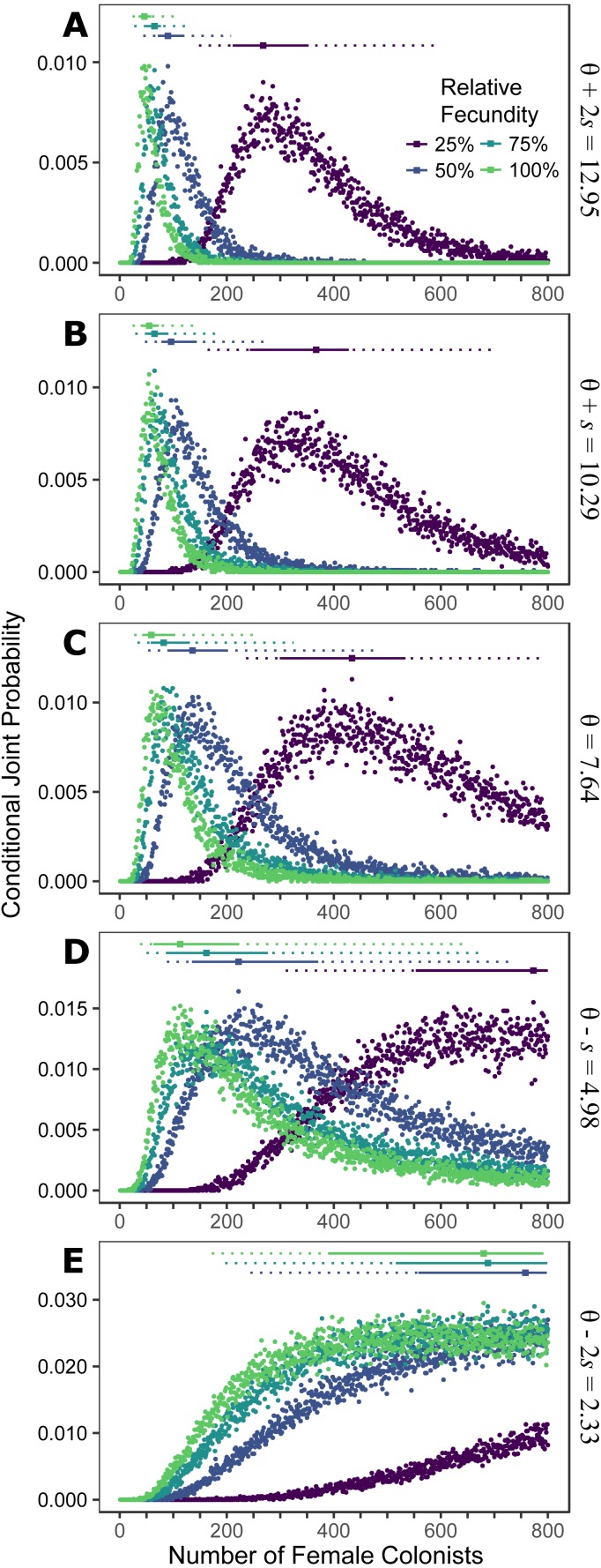
Colonization of western North Atlantic from the Indo-Pacific. The conditional joint probability of simulations resulting in the haplotype richness and diversity observed in the invasive *Pterois volitans* population is plotted against the number of colonizing females. (A–E) represents a different estimated value of *θ* for the source population, ranging from (A) *θ* + 2*s* to (E) *θ* − 2*s*, where *θ* = 7.64 and *s* = 2.73 (standard deviation) were estimated from Indonesian *P. volitans*. Solid and dashed vertical bars above the plots represent the 50% and 95% HPD intervals, respectively. The square point along the HPD line indicates the point estimate of the most likely number of females to have generated the observed pattern of genetic diversity in the invasive population of lionfish. Note that for the lowest value of *θ* (E), the most likely number of female colonists cannot be calculated and the HPD intervals will be skewed low. The same is true for the lowest relative fecundity (25%, purple) in (B–E). This does not affect conclusions because if these parameters describe the source (*θ*) and colonizing populations (relative fecundity), the most probably number of colonists would be much larger than the conservative estimates discussed in the text, and one premise of this effort is that more lionfish colonized the Atlantic than is presently appreciated.

**Figure 2:**
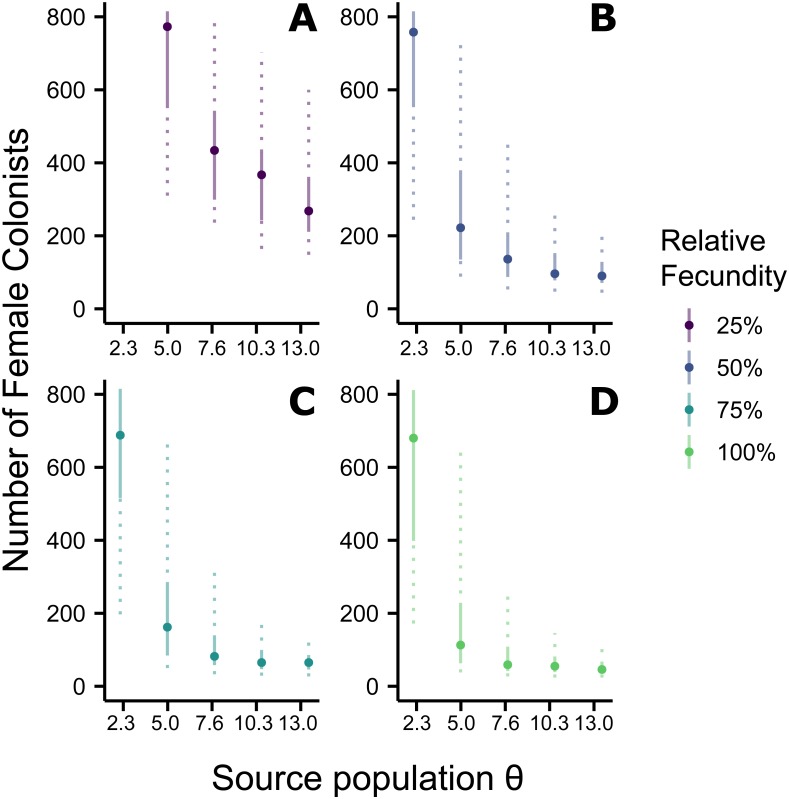
Effect of *θ* on the number of western North Atlantic female colonists. The most likely number of Atlantic *Pterois volitans* female colonists is plotted against *θ* of the source population. Solid and dashed vertical bars represent the 50% and 95% HPD intervals, respectively. Colors indicate the relative fecundity of individual female lionfish in the model. Note that for (A) the most likely number of female colonists at *θ* − 2*s* (2.3) is not shown because it is much greater than 800. The important pattern to recognize is that the number of colonists levels off as *θ* increases, suggesting a lower limit to the number of colonists.

**Figure 3:**
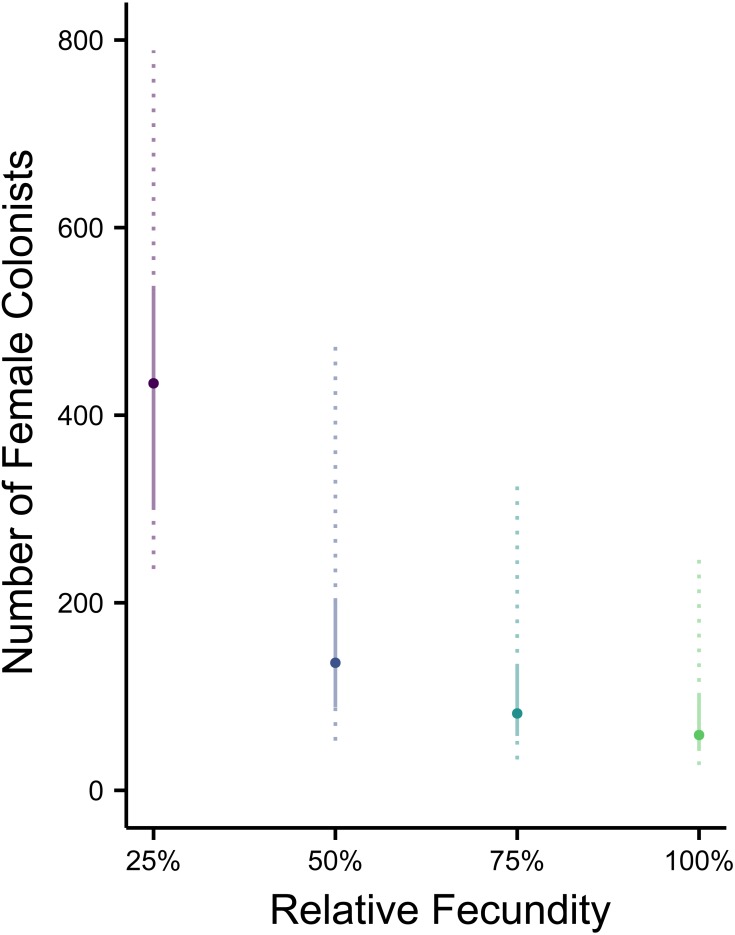
Impact of Allee effects on the number of western North Atlantic female colonists. The most likely number of Atlantic *Pterois volitans* female colonists is plotted against their relative fecundity. The source population depicted has the same *θ* (7.64) as estimated from a population of Indonesian *P. volitans.* Solid and dashed vertical bars represent the 50% and 95% HPD intervals, respectively.

Assuming the *θ* of the source population is the same as the Indonesian population and no Allee effects slowed initial population growth, the most likely number of *P. volitans* females to have colonized the western North Atlantic was 59 (27–257 95% HPD, Fig. 1C). Varying both the diversity of the source population (*θ*) and the strength of Allee effects (fecundity) in the colonizing population strongly influenced the estimate of the number of colonizing lionfish (Figs. 1–3). Increasing the source population diversity and reducing the strength of Allee effects (by increasing fecundity) both resulted in strong exponential declines in the estimate of the number of colonists (Figs. 2 and 3), with an apparent asymptote of ∼44 female colonists (Fig. 2D) in the simulated scenario with no Allee effects and a much more diverse source population (*θ* + 2*s* = 13). The upper bounds of the estimates were much more strongly affected than the lower bounds, indicating that regardless of the diversity of the source population(s) and strength of Allee effects, the lower bounds are highly restrained by general population genetic dynamics. Accounting for Allee effects (50% reduction in fecundity) and the additional possibility that a more diverse population in the Indo-Pacific colonized the Atlantic increased the estimate of introduced female lionfish to ∼90 (44–208, 95% HPD; Fig. 1E).

**Figure 4:**
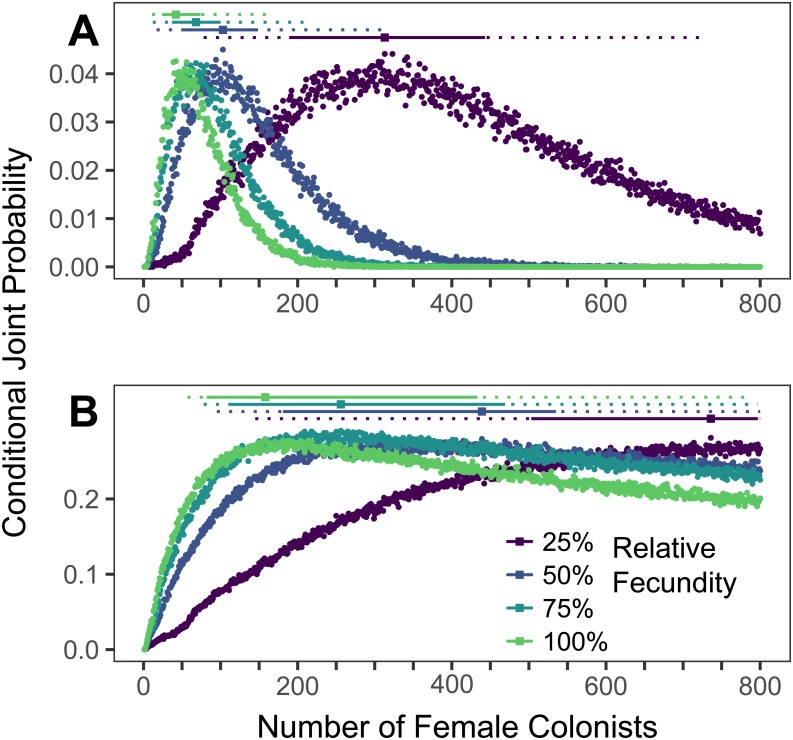
Range expansions from the western North Atlantic to the Caribbean to the Gulf of Mexico. The conditional joint probability of simulations resulting in the haplotype richness and diversity observed in the invasive *Pterois volitans* population is plotted against the number of colonizing females (A) from the western North Atlantic into the Caribbean and (B) from the Caribbean into the Gulf of Mexico. Each point represents 10,000 simulations. Solid and dashed vertical bars above the plots represent the 50% and 95% HPD intervals, respectively. The square point along the HPD line indicates the point estimate of the most likely number of females to have generated the observed pattern of genetic diversity. While more than 800 colonists will need to be simulated to capture the full suite of likelihoods when the relative fecundity is 25%, the rapid growth of the lionfish population in the Caribbean and Gulf suggest that the fecundity of the colonists was not low.

### Simulations of Caribbean and Gulf of Mexico colonizations

The most likely number of *P. volitans* females to have colonized the Caribbean from the western North Atlantic is 42 (11–164 95% HPD, Fig. 4A). The most likely number of females to colonize the Gulf of Mexico from the Caribbean was 158 (57–>800 95% HPD, Fig. 4B), but that was only able to be estimated when the simulation included the maximum fecundity. The lack of an upper bound on the number of Gulf colonists, which is partially due to only simulating up to 800 colonizing females, is consistent with the low diversity of the Caribbean population and its rapid colonization and expansion throughout the Gulf of Mexico. Notably, the simulation results are congruent with previous research showing strong genetic differentiation between the western North Atlantic and Caribbean, and relatively weak genetic differentiation between the Caribbean and Gulf of Mexico (Johnson et al., 2016).

## Discussion

### Colonization of the western North Atlantic from the Indo-Pacific

The simulations of the lionfish invasion performed here suggest that many more than nine female lionfish colonized the Atlantic Ocean. Among all simulations with nine female lionfish colonizing the Atlantic, not one produced the observed haplotype richness and diversity (within the observed 95% confidence interval). Thus, while not impossible, it is exceedingly unlikely that nine female lionfish colonized the Atlantic. The probability that only nine female lionfish colonized the Atlantic might be greater if the source population were characterized by a much greater *θ* than simulated here (≫13). However, the *θ* of the lionfish population in Indonesia is large, and Indonesia occupies a large portion of the range of *P. volitans* (Schultz, 1986; Wilcox et al., 2017). For perspective, we can convert *θ* into the effective population size using a rough estimate of the mutation rate, which is reported to be between approximately 1 × 10^−6^ and 1 × 10^−9^ in humans and fishes (Brown, Beckenbach & Smith, 1993; Parsons et al., 1997; McMillan & Palumbi, 1997; Liu et al., 2006; Castro et al., 2007). For *θ* = 13, the upper estimate and largest value simulated, the effective population size of female lionfish is 6.5 × 10^6^ to 6.5 × 10^9^. Further, the effective population sizes of marine fishes are typically several orders of magnitude lower than the census population size (Hare et al., 2011). Given that the number of colonists levels off with increasing values of *θ* in the source population (Fig. 2), it is unlikely that the source population of lionfish that colonized the Atlantic would be so large as to cause nine female colonists to be a likely scenario. This asymptotic behavior occurs because the nine *P. volitans* haplotypes in the western North Atlantic are not equifrequent and greater than nine colonists are more likely to result in the observed frequencies given realistic genetic compositions of the source population. Ultimately, due to the unequal frequencies of the nine haplotypes, there are effectively no scenarios where nine female colonists are more probable than a larger number of colonists.

If the simulation, is parameterized to minimize the number of colonists (high fecundity and high *θ*), a conservative, minimum estimate of the number of colonists is 24 female lionfish (lower bound of 95% HPD). Other assumptions inherent in the design of the simulation were also made to minimize the colonizing population size, e.g., all females were introduced in one event, no Allee effects, and equal reproductive success. Even so, the most likely number of female colonists with these conservative parameters is 48, with an upper bound of 108 (Fig. 1A).

As the estimated number of female colonists increases, it becomes increasingly improbable (7 × 10^−7^ for 24 female colonists based on the binomial distribution) for only one male to have colonized the Atlantic given the ∼1:1 sex ratio observed in wild populations (Fogg et al., 2013; Downey-Wall, 2016). Therefore, given the range of estimates for the number of female colonists presented here, the most likely number of colonizing males is the same as the number of colonizing females, and all further discussion of the number of colonists will double the estimated number of females to include both sexes. Thus, it is unlikely that fewer than 2 × 24 = 48 lionfish established the Atlantic population. It is very likely, however, that the number of colonizing lionfish was greater than 48.

Making parameters in the model more realistic increases the estimates of the number of colonizing lionfish. For example, slower initial population growth rates due to Allee effects are suggested by the lag time between the detection of lionfish and their spread (Schofield, 2009; Morris & Akins, 2009). In the simulation, the most probable number of colonists increased with slower population growth rates caused by low fecundity. If we assume a 50% reduction in fecundity and the estimated value of *θ* for the Indonesian population, then 272 (106–950, 95% HPD) female and male colonists are predicted (Figs. 1C and 2B). If we were to further allow variation in reproductive success among females, while not simulated, it would depress the genetic diversity of the established population for a given number of colonists (see Hedgecock, 1994). In other words, if variation in reproductive success were introduced in the simulation, then more colonists would be required to generate the observed pattern of genetic diversity in the western North Atlantic.

While most of the assumptions we make result in a conservative estimate for the number of colonists, if multiple isolated source populations colonized the western North Atlantic simultaneously, then the upper bounds for the number of colonists could be lower than estimated here. If there were two source populations with equal contributions of colonists and no shared haplotypes, then the effective number of haplotypes (a measure of diversity that can be easily converted to haplotype diversity, see Jost, 2008) for the colonists, would be the sum of the effective number of haplotypes contributed from each source population. If the same number of total colonists were derived from a single source population with the same diversity, then the most likely effective number of haplotypes could be lower. This effect, however, becomes negligible for small numbers of colonists because the probability of drawing a colonist with a haplotype that has not been drawn previously is similar whether there are one or two source populations, especially given that there are $ \frac{1}{1-0.97} =33$ effective haplotypes in the Indonesian population. Thus, the lower bounds of our estimates are much less likely to be affected if more than one source population of *P. volitans* colonized the western North Atlantic.

### Colonization of the Caribbean and Gulf of Mexico

As the colonizing lionfish population became established, the invasion spread beyond the western North Atlantic, into the Caribbean and later the Gulf of Mexico (Johnson et al., 2016). Distinct genetic structure developed among these regions, roughly matching patterns of genetic structure in other species with a similar range (Taylor & Hellberg, 2006). As we have demonstrated here, each expansion of lionfish across a semipermeable biogeographic barrier can be simulated as a new colonization event, and it is most probable that ∼84 lionfish established the Caribbean population (Fig. 4A).

Due to the small founding population size of the Caribbean, there is an opportunity for genetic drift to cause shifts in genetic composition that perpetuate as the population grows, with a minimal influx of migrants from the founding population (Excoffier & Ray, 2008). This process, termed gene surfing (Hallatschek et al., 2007; Hallatschek & Nelson, 2008), has been observed in bacterial colonies experiencing a range expansion in laboratory settings and predicts that rapidly expanding populations would be characterized by geneticdiscontinuities, as observed in Atlantic-Caribbean lionfish. First reported by Johnson et al. (2016) for lionfish, to our knowledge, this is the first documentation of gene surfing in a wild population. Given the relatively small number of colonists likely to have spread from the western North Atlantic into the Caribbean (84, Fig. 4A), the most parsimonious explanation for this expansion and shift in genetic composition is simply a range expansion coupled with the phenomena of gene surfing, rather than a secondary introduction as posited by Butterfield et al. ( 2015; see Johnson et al., 2016).

The ‘tsunami’ of lionfish that rapidly propagated from the Caribbean was unlikely to have experienced an Allee effect or to promote much additional gene surfing. Indeed, we were unable to precisely estimate the number of colonists initiating the range expansion from the Caribbean into the Gulf of Mexico (Fig. 4B), but it is likely that there were at least 114 colonists (Fig. 4B) to produce the observed pattern of genetic diversity. This lack of convergence suggests that while there is a genetic break between the Caribbean and Gulf of Mexico, it is a relatively permeable barrier, which is supported by conventional population genetic studies and larval tracking work (Johnson et al., 2016; Kitchens et al., 2017). We propose that the vast array of oil platforms in the Gulf of Mexico likely provided ample habitat and aided in the rapid establishment and spread of lionfish throughout the Gulf of Mexico (*sensu* Sheehy & Vik, 2010). It is also important to note that our estimates of the number of initial colonists introduced to each region increases with each additional step. An increasing number of founders in each region aligns with observations of rapid establishment and spread of lionfish as they became introduced to the Caribbean and Gulf of Mexico.

### Additional model assumptions

All models require making assumptions, and the most critical assumptions made in this effort are that (1) *P. volitans* in the Atlantic Ocean originated from a single population in the Indo-Pacific, (2) the Indo-Pacific source population is at or near equilibrium, (3) the mitochondrial control region conforms of the infinite alleles or sites mutational models, (4) the demographic model is reasonably parameterized, (5) a single colonization event led to the establishment of the populations, (6) colonists were adults, and (7) females had equal reproductive success. Several of these assumptions have already been addressed above, but a few require additional discussion. For example, assuming adult lionfish were released minimizes the number of colonists required to establish a population because adults are less likely to die before reproducing than larvae or juveniles.

With respect to the demographic parameters, the model we employed was designed and parameterized for established lionfish populations, but it is likely that Allee effects would result in slower population growth rates. Consequently, we varied fecundity and found that Allee effects would require even more introductions to explain the observed pattern of genetic diversity in the Atlantic. We expect the same to be true for other demographic modifications made to better approximate the natural processes occurring during the early invasion.

A core assumption of the colonization of the western Atlantic is that *P. volitans* is a species. Indeed, we chose to simulate only *P. volitans* because it seems to be the primary invader, with *P. miles* having a smaller invasive footprint. Wilcox et al. (2017) suggest that the Atlantic has been invaded by hybrids formed from *P. miles* and *P. russelii*. Regardless whether the lionfish in the Atlantic are hybrids or not, the results presented here will not qualitatively change. If we were to treat *P. miles* as the same hybrid species as *P. volitans*, then the number of colonizing lionfish would increase with the added genetic diversity in the invading population.

### Propagule pressure and invasions

Propagule pressure, the number and frequency of introductions (Lockwood, Cassey & Blackburn, 2005), is highly correlated with the establishment of alien invasive populations, and it is likely that the Atlantic was subject to substantial propagule pressure from lionfish. In the context of propagule pressure, there are generally two patterns of introduction prior to establishment: (1) sporadic, infrequent introductions of large numbers of individuals, or (2) frequent introductions of small numbers of individuals (see Simberloff, 2009 for both supporting examples and exceptions). Previous invasions that have been triggered by the infrequent introduction of many individuals often involve the intentional release of a species to serve some purpose, after which it becomes a nuisance species (e.g., cane toads throughout the Caribbean and Pacific: Easteal, 1981; various fishes in Hawai’i: Randall, 1987; Gaither et al., 2010). Unintentional large-scale releases are also known to have occurred as a result of escapes from both (1) aquaculture and farming (Naylor, Williams & Strong, 2001; Zapiola et al., 2008; Ramírez et al., 2015) and (2) international shipping in ballast water (Lavoie, Smith & Ruiz, 1999; Drake & Lodge, 2004).

The alternate pathway to the establishment of an alien species, where small introductions occur with relatively high frequency have led to the successful establishment of several alien species (Eurasian house sparrows in North America, European red deer in New Zealand; biocontrol insects in Canada: in Simberloff, 2009). In aquatic systems, introductions are typically attributed to the international aquarium trade and many individuals releasing pets into the environment (Padilla & Williams, 2004; Duggan, Rixon & MacIsaac, 2006; Holmberg et al., 2015; Rhyne et al., 2017). These releases may occur when people, seeking a seemingly more humane option than euthanasia, release unwanted pets into the environment (Courtenay & Taylor, 1986; Duggan, Rixon & MacIsaac, 2006).

### Most likely introduction vector

While many species can survive in ballast water and be transported by shipping traffic (Medcof, 1975; Carlton, 1985), the introduction of lionfish to the Atlantic via ballast water is less likely than through the aquarium trade (Whitfield et al., 2002), and the results presented here reinforce that. In an analysis of international shipping patterns, ornamental marine fish imports, and established populations of alien fishes, Semmens et al. (2004) conclude that southeast Florida reefs (the epicenter of the Atlantic lionfish invasion) host an unusually high number of alien species due to aquarium releases rather than ballast water released by ships. Indeed, lionfish introduced through the aquarium trade are hardy adults that are likely to survive (Wabnitz, 2003). In contrast, fragile larvae have a high probability of mortality upon entering bilge tanks (Gollasch et al., 2000), during transport (Gollasch et al., 2000; Ghabooli et al., 2016) and after release (Padilla & Williams, 2004). Lionfish egg masses float for ∼36–72 h prior to hatching (Fishelson, 1975; Morris, 2009) and are unlikely to enter bilge tanks while floating. Whilst larvae of marine fishes tend to be too large to pass through ballast tank screens (Carlton & Geller, 1993), supposing lionfish larvae did successfully enter the bilge, they would then have to survive travel for approximately 26 days between the Indo-Pacific region and Miami, given an average speed of 24 knots (Notteboom & Cariou, 2009) and two days to traverse the Panama Canal. Larvae begin feeding four days post hatch, can survive six days of starvation (Thresher, 1984), and thus, must feed in the bilge for at least 16 days on depleted plankton populations to survive the voyage (see Gollasch et al., 2000; Ghabooli et al., 2016). A population of lionfish in the bilge would experience an extreme bottleneck and reduced genetic diversity (Ghabooli et al., 2016). If the population does not perish during transport, the larvae must accrue enough resources to metamorphose 20–35 days after fertilization (Ahrenholz & Morris, 2010) and survive for another 11 months before reaching reproductive maturity in the Atlantic (Thresher, 1984). Finally, they must find a mate to propagate the species in their new environment.

If ballast water were the primary vector for the lionfish invasion, then we expect that there would have been several successful introductions in other western Atlantic ports between New York and Brazil, and along the Pacific coast of Mexico (see MacIsaac et al., 2016). It is clear, however, that the lionfish invasion began in one location (near Ft. Lauderdale or Miami, Florida) in the mid-1980s, and all genetic evidence indicates that there were not successful introductions in other locations in the Americas (Johnson et al., 2016). Further, given the results of the simulations presented here, the offspring of at least 24 mothers, and likely many more, would have to have been loaded into ballast tanks, survived the voyage and developed into reproductively mature adults that successfully reproduced. Adult lionfish have been found at densities up to 26.3 ha^−1^ in the Indo-Pacific region (Kulbicki et al., 2012); up to ∼3,000 m^3^ of ballast water is released by a ship (MacIsaac et al., 2016) originating from a port in the native range of lionfish; and thus, 0.33 m^3^ of ballast water per m^2^ of lionfish habitat across at least one hectare is required to transport the requisite genetic diversity to have triggered the invasion. At sea, only small volumes of ballast water are taken on for trimming the ship. While in port, ships generally take on ballast while docked, and water can be pumped at rates of 1,000–2,000 m^3^ hr^−1^ on container ships and up to 20,000 m^3^ hr^−1^ on tankers (Board & Council, 1996). Consequently, even in an unusual port with strong currents, it seems unlikely that one ship would take on all of the genetic diversity contained in one hectare of saturated lionfish habitat in the Indo-Pacific region. Regardless, many ships, would have transported lionfish larvae to many ports in the western Atlantic with viable lionfish habitat, and yet lionfish only established in Florida and spread from there. The ballast hypothesis does not hold water.

We conclude, as others have previously, that the aquarium trade was the most likely vector for the lionfish invasion (Hare & Whitfield, 2003; Semmens et al., 2004; Ruiz-Carus et al., 2006; Morris & Whitfield, 2009). In light of the ∼180 colonizing lionfish estimated here, it seems most parsimonious to conclude that a combination of small and larger releases caused the lionfish invasion. If the colonization involved several introductions of a small number of fish by home aquarists, then many more lionfish would have to be released to generate the observed pattern of genetic diversity in the Atlantic Ocean. Lionfish are voracious predators and are prime candidates to be a nuisance by consuming other fish in a tank; thus, it is not implausible that many aquarists would discard lionfish from their aquaria in the 1970s and 1980s when the home saltwater aquarium trade skyrocketed (Andrews, 1990; Chapman et al., 1997). Indeed, there is at least one documented case of an unintentional release from a private aquarium in Miami, FL in 1992 caused by Hurricane Andrew (Courtenay, 1995). Lionfish from piecemeal releases, however, would need to be in the same locations (such as ports, harbors or piers) or locate each other and aggregate after introduction. Consequently, it is plausible or even likely that importers, intermediaries, and aquarists have released lionfish into the Atlantic, leading to the establishment of the population. For example, if a distributor were to go out of business, they might choose to release the fish, rather than transferring ownership of the fish or euthanizing them.

### Preventing future invasions

Propagule pressure is an important predictor of invasion success (Lockwood, Cassey & Blackburn, 2005), and despite the destructive consequences of the lionfish invasion, there is still a healthy market serviced by the port of Miami. Between 2008 and 2011 approximately 7,000 ± 3,000 (SD) *Pterois* spp. yr^−1^ were imported into the port of Miami, the site of the earliest reports of lionfish in the Atlantic (Schofield, 2009). Approximately 70% were visually-identified as *P. volitans* (Rhyne et al., 2017; http://www.aquariumtradedata.org), meaning that 30% were other lionfish species that might become established if introduced. There are several other species of lionfish in the Indo-Pacific region, including one, *Pterois miles*, that has also established a population in the western North Atlantic (Hamner, Freshwater & Whitfield, 2007). Hybridization of other *Pterois* spp. with the existing invasive lionfish population is also possible (see Wilcox et al., 2017), providing a potential adaptive subsidy to the invasive population. A small fraction of the lionfish or other species being imported into Miami, FL, and other locations annually could trigger another invasion.

Many invasions have been triggered by very small numbers of individuals (Simberloff, 2009); therefore, management and enforcement should act to safeguard against a similar invasion in the future by eliminating propagule pressure (Caffrey et al., 2014). One focus should be on containment of live alien species to minimize the risk of an accidental large-scale release of potential invaders. A second focus should be on educating wholesalers, retailers, home aquarists, and the public about the dangers to the ecosystem and economy of releasing unwanted pets into the environment. Release into the wild is potentially viewed as a favorable alternative to euthanasia for an unwanted pet, therefore, this education effort could be coupled with some form of buy-back program where unwanted pets can be traded back to a store or government agency as a humane and ecosystem-friendly option (Courtenay & Taylor, 1986; Courtenay & Stauffer, 1990; Duggan, Rixon & MacIsaac, 2006). A third focus should be on implementing penalties for violating regulations and incentives for following them to increase compliance (Furlong, 1991; Keane et al., 2008; Floerl, Inglis & Diettrich, 2016). Game theory dictates that cheating is a viable strategy, unless a combination of the (1) risk of getting caught and (2) the cost associated with getting caught is sufficiently great (Smith, 1982). If regulatory entities and aquarium trade stakeholders take the threat of biological invasions seriously, it should be possible to avoid another devastating invasion like the lionfish in the Atlantic.

##  Supplemental Information

10.7717/peerj.3996/supp-1 Supplemental Information 1 Supplemental code Two R code files are included the first: “model_functions.R” contains the code used to run the model as described here. The second “run_lionfish_colonizations.R” initializes the relevant data collected from the literature as described in the manuscript and runs the model for different source and destination populations.

10.7717/peerj.3996/supp-2 Supplemental Information 2 Supplemental raw data—FASTA This file contains mtDNA sequence data from the control region of *Pterois volitans* in the FASTA file format. Samples were obtained from Indonesia, the western North Atlantic, Caribbean, and Gulf of Mexico via GenBank (Freshwater et al., 2009; Betancur et al., 2011; Toledo-Hernández et al., 2014; Butterfield et al., 2015; Johnson et al., 2016).

10.7717/peerj.3996/supp-3 Supplemental Information 3 Supplemental raw data—Arlequin This file contains mtDNA sequence data from the control region of *Pterois volitans* in the Arelquin file format. Samples were obtained from Indonesia, the western North Atlantic, Caribbean, and Gulf of Mexico via GenBank (Freshwater et al., 2009; Betancur et al., 2011; Toledo-Hernández et al., 2014; Butterfield et al., 2015; Johnson et al., 2016).

## Additional Information and Declarations

### Data Availability

The following information was supplied regarding data availability: Two R code files have been provided as Supplemental Files.
